# High-Flow Nasal Cannula in Weaning Patients from Mechanical Ventilation in Head and Neck Surgery: Retrospective Study

**DOI:** 10.3390/life15081264

**Published:** 2025-08-10

**Authors:** Vincenzo Pota, Francesco Coppolino, Luca Gregorio Giaccari, Manlio Barbarisi, Marco Fiore, Mario Santagata, Maria Beatrice Passavanti, Maria Caterina Pace, Luigi Rugge, Gianpaolo Tartaro, Pasquale Sansone, Caterina Aurilio

**Affiliations:** 1 Department of Women, Child, General and Specialistic Surgery, University of Campania “L. Vanvitelli”, 80138 Naples, Italycaterina.aurilio@unicampania.it (C.A.); 2 Vito Fazzi Hospital, 73100 Lecce, Italy; 3 Multidisciplinary Department of Medical-Surgical and Dental Specialties, University of Campania “L. Vanvitelli”, 80138 Naples, Italy

**Keywords:** HFNC, postoperative hypoxemia, head and neck surgery

## Abstract

**Background:** Patients undergoing head and neck surgery with free flap reconstruction are at a high risk for postoperative respiratory complications, including hypoxemia. Conventional oxygen therapy (COT) and non-invasive ventilation (NIV) may be poorly tolerated or contraindicated due to anatomical limitations. High-Flow Nasal Cannula (HFNC) therapy represents a promising alternative, offering better humidification, comfort, and oxygenation. **Methods:** This retrospective single-center study included 50 adult patients admitted to the ICU after head and neck oncologic surgery with flap reconstruction from January 2022 to November 2024. All patients received HFNC immediately after extubation. Hypoxemia was defined as a PaO_2_/FiO_2_ (P/F) ratio of < 300 mm Hg. The primary outcome was the incidence of postoperative hypoxemia. Secondary outcomes included reintubation rates and patient compliance. Data were collected at 1, 6, 12, and 24 h following HFNC initiation. **Results:** Out of 59 patients screened, 9 were excluded per predefined criteria. Among the 50 included, only 2 patients (4%) developed hypoxemia, with P/F ratios remaining above 250. No patients required reintubation. The respiratory rate–oxygenation index (ROX index) improved steadily during the first 24 h. HFNC was well tolerated; only three patients required minor adjustments due to discomfort. **Conclusions:** HFNC use in the immediate postoperative period after head and neck surgery was associated with a low incidence of hypoxemia and no reintubations. These findings suggest that HFNC is a safe and effective strategy for postoperative respiratory support in this high-risk population. Further prospective studies are warranted to confirm the benefit of HFNC in reducing hypoxemia and preventing reintubation in high-risk surgical populations.

## 1. Introduction

Postoperative pulmonary complications (PPCs), including hypoxemia, represent a major cause of morbidity and mortality following major surgery, especially in procedures involving the head and neck region. The global prevalence of postoperative hypoxemia varies significantly but is estimated around 16–20% according to recent meta-analyzes, with rates as high as 33% in low-income countries and high-risk populations [1].

Hypoxemia is driven by a variety of factors, including reduced lung volumes, diaphragmatic dysfunction, impaired ventilation–perfusion ratios, and atelectasis formation [2]. These respiratory alterations are exacerbated in patients undergoing major head and neck surgeries due to anatomical complexity, the surgical duration, and postoperative airway compromise.

In recent years, High-Flow Nasal Cannula (HFNC) has emerged as a promising non-invasive respiratory support technique, delivering warmed, humidified oxygen at high flow rates, improving gas exchange and patient comfort. Unlike conventional oxygen therapy (COT), HFNC offers low-level positive pressure, reduces the anatomical dead space, and improves oxygenation even in hypoxemic acute respiratory failure. Several clinical reviews and expert consensus guidelines now support the use of HFNC over COT or NIV in selected surgical populations for both preventive and therapeutic purposes [3,4,5]. Managing ventilation in patients with postoperative hypoxemia is challenging due to the type of interface required for oxygen therapy delivery. Oxygen therapy (using a nasal cannula or a mask) and non-invasive positive pressure ventilation (NPPV) are widely used as sequential treatments after weaning from mechanical ventilation (MV) in hypoxemic patients. NPPV can be complex to implement, offering lower comfort and tolerance, with a high incidence of complications such as reflux and aspiration. This limits its application, especially in the context of maxillofacial surgery, where NPPV is often contraindicated [3,4,5,6].

Head and neck surgeries for cancerous pathologies have steadily increased in recent years [7]. Due to the complexity and esthetic importance of anatomical structures in the head and neck region, reconstruction with free tissue transfer is often required to achieve optimal postoperative function and appearance [8]. According to the Enhanced Recovery After Surgery (ERAS) protocol for patients undergoing head and neck cancer surgery, key elements during the postoperative phase in the Intensive Care Unit (ICU) include airway management and pain control [9].

Since January 2022, we began treating postoperative hypoxia in patients undergoing head and neck surgery with free tissue transfer reconstruction using HFNC (HFNC) as part of routine clinical practice. This retrospective study analyzed registry data before and after updating our postoperative protocol for managing hypoxia in ICU-admitted patients following head and neck surgery.

## 2. Methods

Data collection for 50 patients was conducted in accordance with the Declaration of Helsinki and Good Clinical Practice guidelines. All patients provided written informed consent for data collection. As per the institutional policy, informed consent was obtained from all patients preoperatively for the use of anonymized data in future research.

All participant data were protected against coercion, and confidentiality was strictly maintained. We analyzed patients from the Intensive Care Unit of the University Hospital of Campania “L. Vanvitelli” from January 2022 to November 2024. We obtained CE approval (AOU Università della Campania Vanvitelli—Prot. 0014496/i).

The inclusion criteria were as follows:

- Patients extubated after head and neck cancer ablation with concomitant free flap reconstruction surgery and admitted to the ICU.
- Application of HFNC.
- Adults over 18 years of age who were able to provide consent.
- Exclusion criteria included the following:
- Chronic hypoxiemic disease.
- Need of non-invasive ventilation during the preoperative period.

Extubation was scheduled following a period of ventilatory stabilization and complete weaning with pressure support ventilation (PSV). All patients were extubated under sedation with a continuous infusion of dexmedetomidine at 0.6–1.2 mcg/kg/h. Extubation was followed by HFNC set at 37 °C with a flow rate ranging from 50 to 60 L/min. FiO_2_ level were adjusted to maintain a target peripheral oxygen saturation (SpO_2_) of ≥95%.

We collected clinical data, comorbidities, body mass index (BMI), biochemical blood analysis, arterial blood gas concentrations (upon HFNC initiation and at subsequent measurements), ventilatory support settings for each patient, and the ROX Index. The ROX (respiratory rate–oxygenation) index, calculated as SpO_2_/FiO_2_ divided by the respiratory rate (RR), was utilized as a non-invasive, dynamic marker of respiratory performance following extubation. This index provides a composite measure reflecting both oxygenation status and ventilatory demand, allowing clinicians to monitor the effectiveness of High-Flow Nasal Cannula (HFNC) therapy over time. In this study, ROX values were recorded at 1, 6, 12, and 24 h after HFNC initiation to assess trends in respiratory function and identify early signs of clinical improvement or deterioration.

Data were recorded in an electronic database. The primary endpoints were to evaluate changes in the incidence of desaturation and the need for reintubation. The secondary endpoint was patient compliance with the HFNC device.

Sequential data were collected at 1, 6, 12, and 24 h after HFNC treatment initiation, including the ratio of arterial oxygen partial pressure (PaO_2_) to fractional inspired oxygen (FiO_2_), known as the P/F ratio, to investigate hypoxemia incidence.

The definition used for hypoxemia was a P/F ratio of <300 mm Hg.

The need for reintubation was evaluated in terms of the ROX index (SpO_2_/FiO_2_/respiratory rate) and the absolute percentage of patients reintubated within the first 24 h.

The absolute risk (AR) of hypoxemia was calculated.

**Figure 1:**
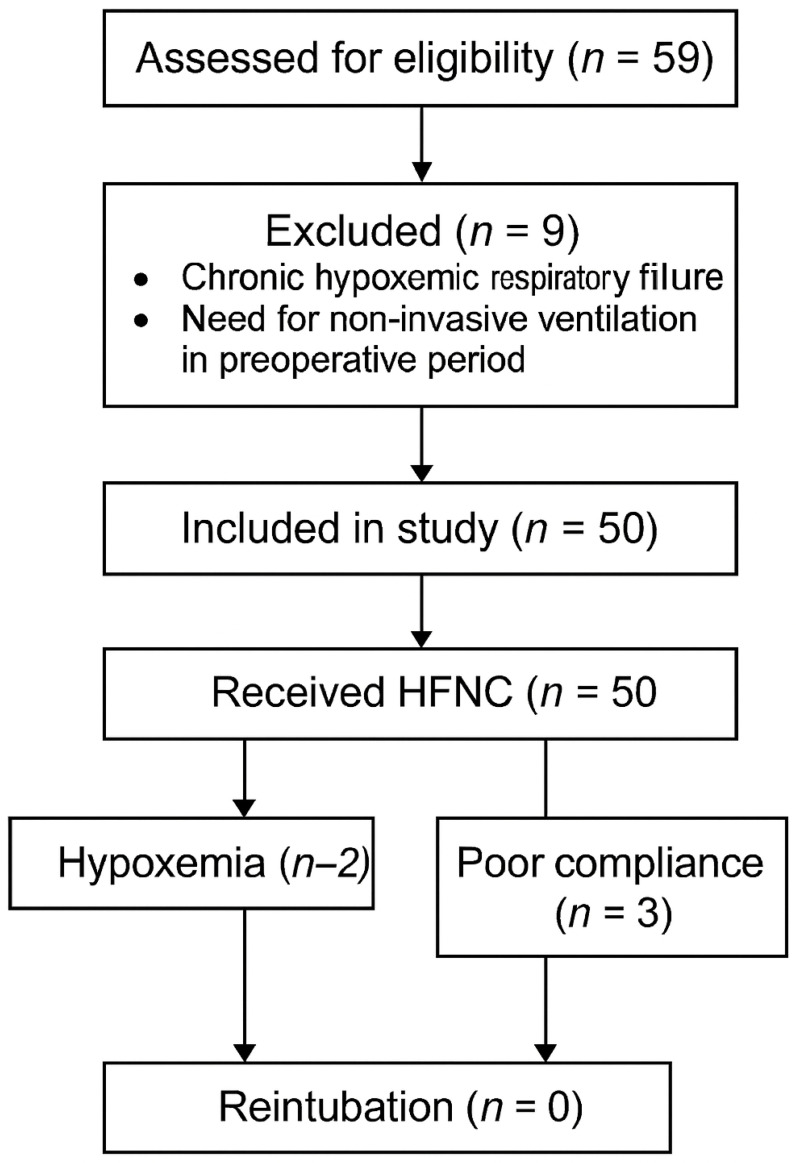
CONSORT flow diagram illustrating the study population screening and inclusion process. A total of 59 patients were assessed for eligibility. Nine patients were excluded due to either chronic hypoxemic respiratory failure or the need for non-invasive ventilation in the preoperative period. Fifty patients met the inclusion criteria and received postoperative respiratory support with High-Flow Nasal Cannula (HFNC). Among them, 2 developed mild hypoxemia, while 3 showed initial poor compliance with HFNC. No patients required reintubation.

The flow chart (Figure 1), according to the CONSORT flow diagram, provides details on patient selection. Out of the 59 medical records reviewed, 9 patients did not meet the inclusion criteria. Fifty patients were included in the study. Data supporting the findings of this article are available in the medical records archive of “Luigi Vanvitelli” University Hospital (Piazza Luigi Miraglia 2—Naples, Italy).

Patients were shifted to oxygen delivery via low-flow nasal cannula after the first 24 h.

The overall incidence of patients remaining on HFNC after the first 24 h were recorded.

## 3. Statistical Analysis

Descriptive statistics were used to summarize patient characteristics and clinical outcomes. Continuous variables, such as ROX index values at 1 h, 6 h, 12 h, and 24 h post-extubation, were expressed as the mean ± standard deviation (SD). The ROX index trend over time was evaluated using aggregated means at each timepoint, without formal hypothesis testing, given the descriptive and observational nature of the study. No inferential statistical tests were applied due to the lack of a comparison group. Data were analyzed using Microsoft Excel (version [indica versione]) for basic statistical computation and visualization. Missing values, if any, were excluded from the analysis.

## 4. Results

Of the 59 patients who underwent surgery during the evaluated period, 9 were excluded due to chronic hypoxemic respiratory failure or other diseases requiring non-invasive ventilation during the preoperative assessment. The clinical data of patients are listed in Table 1.

The clinical characteristics of the included patients revealed a predominance of ASA III classification (100%) and a mean BMI of 27.5 ± 2.1 kg/m^2^. The majority of free flap reconstructions were radial forearm or anterolateral thigh flaps. The mean duration of surgery was 677.5 ± 72.5 min, and the average length of ICU stay was 2.3 ± 0.7 days.

**Figure 2:**
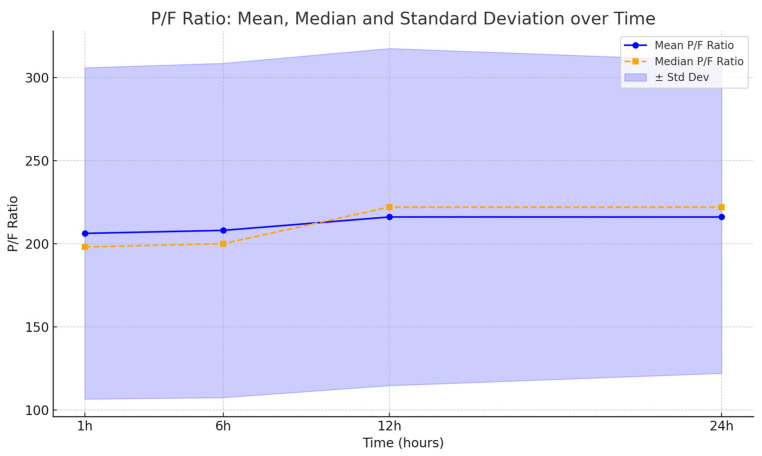
P/F ratio trends at 1 h, 6 h, 12 h and 24 h post-extubation in patients treated with HFNC.

Across all patients treated with HFNC post-extubation, none required escalation to invasive ventilation. All included patients received HFNC (HFNC) oxygen therapy in the postoperative period. Only two patients presented hypoxia, although their P/F ratios remained above 250 (Figure 2). Therefore, the absolute risk (AR) of hypoxemia in the analyzed cohort was 0.04 (4%). Mean P/F ratios remained consistently above 300 in 96% of patients. The reintubation rate was 0%.

**Figure 3:**
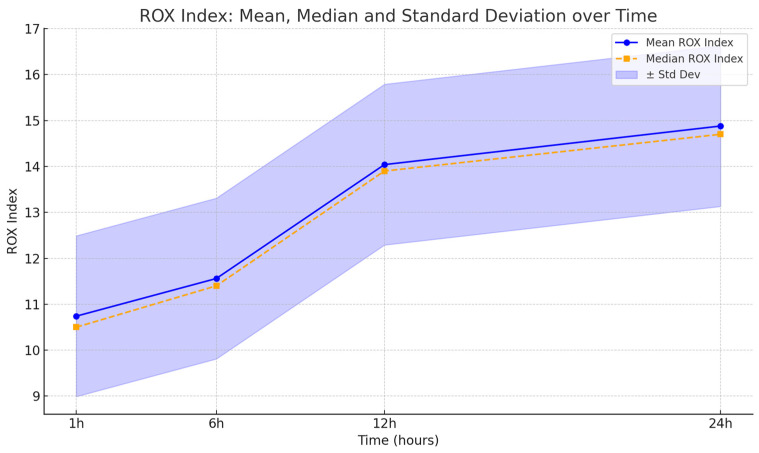
ROX index progression in the first 24 h post-extubation.

The ROX index progressively improved over the first 24 postoperative hours. The mean ± standard deviation values were 10.74 ± 1.7, 11.56 ± 1, 14.04 ± 1, and 14.88 ± 0.98 at 1 h, 6 h, 12 h, and 24 h, respectively (Figure 3).

These values are in line with predictors of success (>4.88 at 12 h), supporting the utility of HFNC in postoperative ICU management. Patient comfort was high: only three patients reported mild nasal dryness, which was resolved by adjusting the gas temperature from 37 °C to 35 °C.

No adverse events such as aspiration pneumonia, bleeding, or unplanned ICU readmissions were recorded. Transition to low-flow oxygen occurred after 24 h in 90% of cases. (see Table 1).

**Figure 4:**
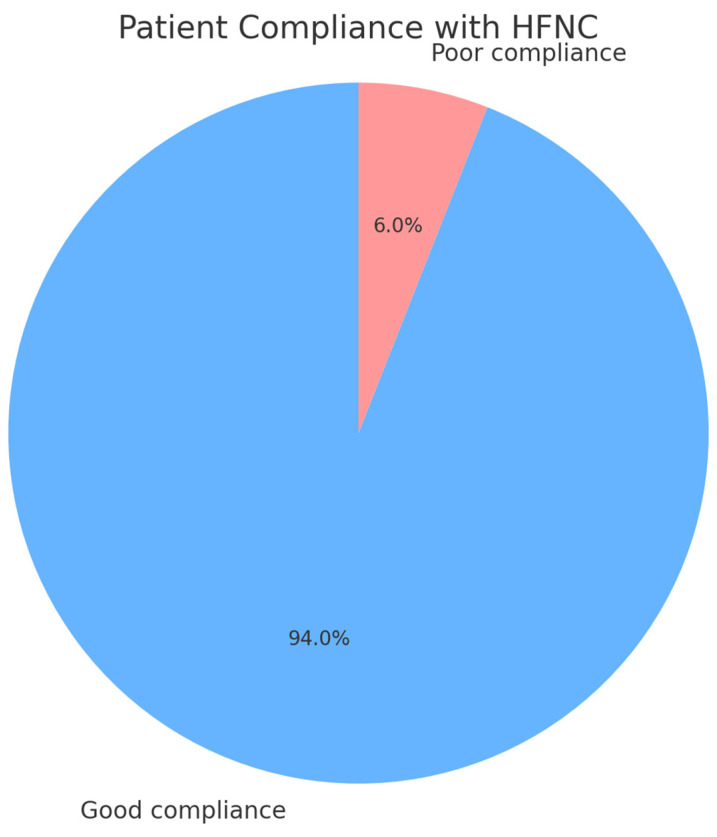
Patient compliance and adjustments required for HFNC therapy.

Regarding compliance and adherence to the device, only three patients showed poor adherence, which were successfully resolved by reducing the temperature from 37 °C to 35 °C (Figure 4).

## 5. Discussion

The findings of our retrospective study align with previous studies indicating the superiority of HFNC in managing postoperative hypoxemia. Vetrugno and Pensier describe HFNC as effective in optimizing oxygenation and reducing respiratory complications in perioperative settings [2,10]. In our cohort, a hypoxemia rate of 4% and zero reintubations were recorded, markedly lower than reported in conventional oxygen therapy cohorts (16–22%). The ROX index increase suggests sustained improvement in ventilation efficiency. The use of dexmedetomidine during extubation may have also contributed to smoother weaning by reducing sympathetic drive and enhancing tolerance. Unlike non-invasive ventilation, which may induce patient self-inflicted lung injury (P-SILI), HFNC provides gentle support with greater comfort, avoiding mask intolerance or pressure ulcers.

A recent network meta-analysis by Li et al. further reinforces the role of HFNC as a preferred strategy in the prevention of postoperative pulmonary complications (PPCs), particularly in obese patients. The study compared CPAP, BiPAP, HFNC, and COT in over 1100 obese surgical patients and found that HFNC was among the most effective interventions for reducing the risk of atelectasis, hypoxemia, and treatment failure while also significantly shortening the length of hospital stay. These findings highlight the potential benefits of HFNC not only in general surgical populations but also in specific subgroups at a higher risk of PPCs, such as obese individuals [11].

Meta-analyses confirm that HFNC reduces escalation needs and shortens ICU stays. Importantly, our study reflects real-world, routine use in ICU protocols, supporting integration of HFNC in postoperative care pathways [1,11,12]. Limitations include its retrospective design and a small sample size, warranting future RCTs to confirm these promising findings.

Postoperative pulmonary complications (PPCs) are generally defined as pulmonary abnormalities that lead to clinically significant disease or dysfunction, which adversely affects the postoperative clinical course. Key PPCs include pneumonia, atelectasia, bronchospasm, exacerbation of chronic obstructive pulmonary disease (COPD), and respiratory failure [13,14,15,16]. The incidence of PPCs has been reported to be approximately 8% among surgical patients, with morbidity and mortality following major surgery reaching nearly 30%

The incidence of postoperative hypoxemia varies in studies [17]. An observational study showed that among 26.7% of patients, hypoxic events were observed for the first 20 min in PACU admission. Which is similar to the finding in this research [18]. Similar to this study, the incidence was 22.7% [19]. Zhang et al. showed that the incidence of hypoxemia at the PACU was 2.79% [20], but there were oxygen supplementation during the patient transfer from the OR to the PACU in their study. Another retrospectively study showed that postoperative desaturation was 12.1%, which was lower than this study despite including only patients who underwent general anesthesia [21]. We have demonstrated that the application of HFNC in the first 24 postoperative hours is able to reduce the incidence of desaturation to 4%. Compared to published data reporting a postoperative hypoxemia incidence ranging from 12.1% to 22.7% in similar surgical populations [18,19,20,21], our observed incidence of 4% under HFNC use suggests a relative risk reduction of approximately 67% to 82%. Although this is an indirect comparison, it highlights the potential clinical benefit of HFNC in this specific context.

HFNC is a noninvasive, high-concentration oxygen delivery system that addresses some of the limitations of COT. By providing airflow rates as high as 50–60 L/min, HFNC closely meets the inspiratory needs of dyspneic patients with AHRF and reliably delivers FiO_2_ as high as 100% while also providing a low level of positive end-expiratory pressure (PEEP) in the upper airways, facilitating alveolar recruitment [22]. While some evidence indicates that HFNC, particularly at high flow rates, generates PEEP near 5 cm H_2_O, simply opening the mouth reduces the PEEP level to lower values (about 1 cm H_2_O), making a net effect or alveolar recruitment unlikely [23].

NIV improves oxygenation by increasing mean airway pressure but has the potential to deliver injurious lung volumes that may put patients at risk for patient self-inflicted lung injury (P-SILI). In contrast, HFNC provides less positive pressure ventilatory support than NIV, which may decrease the risk of P-SILI and can help improve patient tolerance [17]. Other potential benefits of HFNC over COT include avoiding harmful changes in transpulmonary pressure, carbon dioxide cleareance f upper airways, improved ventilation, and provision of reliable humidification, which may result in increased patient comfort and enhanced secretion clearance [24].

The optimal mode of noninvasive respiratory support in AHRF remains unclear. The few trials that have directly compared HFNC to NIV have focused on specific populations in the Intensive Care Unit (ICU). The largest trial found that for ICU patients with pure hypoxemic respiratory failure, HFNC decreased mortality compared with NIV [24]. An other import aspect of the study is the we have reduced the risk of reintubation to zero via the application of HFNC in the first 24 postoperative hours. Among adult patients at a high risk of extubating failure, HFNC is not inferior to NIV in preventing reintubation, mortality, and respiratory failure. It may shorten the length of stay, reduce adverse reactions, and increase patient comfort. However, the heterogeneity of PaCO2, the respiratory rate (RR), and the length of stay is evident. Thus, a high-quality randomized controlled trial (RCT) is required to confirm whether HFNC therapy can provide an advantage in the length of stay and respiratory function in such patients [25].

Patients undergoing major abdominal surgery, a preventive application of HFNC after extubating, compared with COT, did not result in a statistically significant reduction in the incidence of postoperative hypoxemia. The routine use of postoperative HFNC after extubating does not seem to be justified in similar patients [26].

A systematic review and meta-analysis on the routinely use of HFNC in the immediate postoperative period demonstrate that HFNC prevents intubation in acute hypoxic respiratory failure when compared to conventional oxygen therapy. In this study the use of HFNC, compared to COT, significantly decreased the need for reintubation. The rate was 0.9% in the HFNC group and 4.3% in the COT, but this outcome is based on imprecision because the required information size was not met. HFNC use was also associated with a significant decrease in the need for escalation of respiratory support, with very low certainty evidence. Compared to NIV, HFNC showed no difference in the reintubation rate or the rate of respiratory therapy failure [27].

Twose et al. valuated HFNC in patients undergoing major head and neck surgery with tracheostomy and found improved comfort and humidification, with reduced pulmonary complications such as atelectasis and infection, compared to standard oxygen therapy. These findings are particularly relevant to our population, where airway management and secretion clearance are paramount. Together, these studies reinforce the role of HFNC as a preferred strategy in managing post-extubation respiratory support in surgical ICU settings [28].

Additionally, another systematic review involving nine trials showed a consistent reduction in post-extubation failure with HFNC compared to conventional oxygen therapy, with a relative risk of 0.46 (95% CI: 0.30–0.70). Together, these results reinforce the role of HFNC in improving respiratory outcomes and avoiding escalation to invasive support [29].

The lack of a comparative control group limits our ability to directly assess the effectiveness of HFNC. Future prospective studies, ideally with randomization or comparison with historical in-hospital cohorts prior to HFNC implementation, are warranted to validate these findings.

## 6. Conclusions

This retrospective analysis highlights the effectiveness of HFNC (HFNC) therapy in reducing early postoperative hypoxemia and avoiding reintubation in head and neck surgery patients. The progressive improvement in the P/F ratio and the ROX index over the first 24 h, with consistently rising means and medians, indicates enhanced oxygenation and respiratory function. The low incidence of hypoxemia (4%) and the absence of reintubation further validate HFNC as a safe and well-tolerated postoperative support strategy. Despite the lack of formal statistical testing, these results support the integration of HFNC into routine ICU postoperative care for patients undergoing complex oncologic maxillofacial procedures. Future prospective randomized trials are warranted to confirm these findings and explore long-term outcomes.

## 7. Limitation

This study presents several limitations. First, its retrospective observational design without a control group limits the ability to draw causal inferences regarding the effectiveness of HFNC therapy. The absence of a comparative cohort treated with conventional oxygen therapy (COT) or non-invasive ventilation (NIV) precludes a direct estimation of the relative risk within the same population. Although we reported a lower incidence of hypoxemia (4%) compared to rates described in previous studies (ranging from 12% to 22%), this relative risk estimation is based on an indirect comparison and should be interpreted with caution. Additionally, the small sample size and the single-center nature of the study may affect the generalizability of the findings. Further prospective randomized controlled trials are necessary to confirm these results and better quantify the benefit of HFNC in similar surgical populations.

## Figures and Tables

**Table 1:** Clinical data.

ASA	III
Gender	60% Male
	40% Female
**Length of Surgery**	677.5 min (DS 72.5)

## Data Availability

All data are present at the University of Campania “L. Vanvitelli”.

## Abbreviations

AHRF	Acute Hypoxemic Respiratory Failure
AR	Absolute Risk
BMI	Body Mass Index
COT	Conventional Oxygen Therapy
ERAS	Enhanced Recovery After Surgery
FiO_2_	Fraction of Inspired Oxygen
HFNC	High-Flow Nasal Cannula
ICU	Intensive Care Unit
length of stay	Length of Stay
MV	Mechanical Ventilation
NIV	Non-Invasive Ventilation
NPPV	Non-Invasive Positive Pressure Ventilation
OR	Operating Room
PACU	Post-Anesthesia Care Unit
PaO_2_	Arterial Partial Pressure of Oxygen
PEEP	Positive End-Expiratory Pressure
P/F ratio	Ratio of PaO_2_ to FiO_2_
PPC	Postoperative Pulmonary Complication
PSV	Pressure Support Ventilation
RCT	Randomized Controlled Trial
RR	Respiratory Rate
ROX Index	SpO_2_/FiO_2_ to Respiratory Rate Index
SpO_2_	Peripheral Oxygen Saturation
